# Antimicrobial stewardship in Australia: the role of qualitative research in programme development

**DOI:** 10.1093/jacamr/dlab166

**Published:** 2021-11-18

**Authors:** Karin A Thursky, Laura Y Hardefeldt, Arjun Rajkhowa, Courtney Ierano, Jaclyn Bishop, Lesley Hawes, Ruby Biezen, Sajal K Saha, Leslie Dowson, Kirsten E Bailey, Ri Scarborough, Stephen B Little, Fiona Gotterson, Brian Hur, Anna Khanina, Karen Urbancic, Helen K Crabb, Suzanna Richards, Anna Sri, Rodney James, David C M Kong, Caroline Marshall, Danielle Mazza, Trisha Peel, Rhonda L Stuart, Jo-Anne Manski-Nankervis, N Deborah Friedman, Noleen Bennett, Thomas Schulz, Helen Billman-Jacobe, Evette Buono, Leon Worth, Ann Bull, Michael Richards, Darshini Ayton, James R Gilkerson, Glenn F Browning, Kirsty L Buising, National Centre for Antimicrobial Stewardship, Elizabeth Manias, Jason Trubiano, Brendan McMullan, Abby Douglas, Monica Slavin, Mark Davis, Caroline Chen, Robyn Ingram, Ron Cheah, Xin Fang, Jegadeesh Sakthivel, Sonia Koning

**Affiliations:** 1 NHMRC National Centre for Antimicrobial Stewardship, Department of Infectious Diseases, University of Melbourne, 792 Elizabeth Street, Melbourne, Victoria 3000, Australia; 2 Department of Infectious Diseases, Faculty of Medicine, Dentistry and Health Sciences, University of Melbourne, 792 Elizabeth Street, Melbourne, Victoria 3000, Australia; 3 Victorian Infectious Diseases Service, Royal Melbourne Hospital, Melbourne Health, 300 Grattan Street, Parkville, Victoria 3050, Australia; 4 Guidance Group, Royal Melbourne Hospital, Melbourne Health, 792 Elizabeth Street, Melbourne, Victoria 3000, Australia; 5 National Centre for Infections in Cancer, Sir Peter MacCallum Department of Oncology, University of Melbourne and Peter MacCallum Cancer Centre, 305 Grattan Street, Melbourne, Victoria 3000, Australia; 6 Asia-Pacific Centre for Animal Health, Melbourne Veterinary School, Faculty of Veterinary and Agricultural Sciences, University of Melbourne, Corner Park Drive and Flemington Road, Building 400, Parkville, Victoria 3010, Australia; 7 Pharmacy Department, Ballarat Health Services, 1 Drummond Street North, Ballarat, Victoria 3353, Australia; 8 Department of General Practice, Monash University, 1/270 Ferntree Gully Road, Notting Hill, Victoria 3168, Australia; 9 Department of General Practice, University of Melbourne, 780 Elizabeth Street, Melbourne, Victoria 3010, Australia; 10 Centre for Medicine Use and Safety, Faculty of Pharmacy and Pharmaceutical Sciences, Monash University, 381 Royal Parade, Parkville, Victoria 3052, Australia; 11 Department of Infectious Diseases, The Alfred and Central Clinical School, Burnet Institute, Monash University and Alfred Health, 85 Commercial Road, Monash University, Melbourne, Victoria 3004, Australia; 12 Departments of Infectious Diseases and Infection Control and Epidemiology, Monash Medical Centre, Monash Health, 246 Clayton Road, Clayton, Victoria 3168, Australia; 13 Department of Infectious Diseases, University Hospital Geelong, Barwon Health, Bellerine Street, Geelong, Victoria 3220, Australia; 14 VICNISS Coordinating Centre, Melbourne Health, 792 Elizabeth Street, Melbourne, Victoria 3000, Australia; 15 New South Wales Clinical Excellence Commission, 1 Reserve Road, St Leonards, New South Wales 2065, Australia; 16 Department of Epidemiology and Preventive Medicine, Monash University, 553 St Kilda Road, Melbourne, Victoria 3004, Australia; 17 Peter Doherty Institute of Infection and Immunity, 792 Elizabeth Street, Melbourne Victoria, 3000, Australia

## Abstract

Antimicrobial stewardship (AMS) in Australia is supported by a number of factors, including enabling national policies, sectoral clinical governance frameworks and surveillance programmes, clinician-led educational initiatives and health services research. A One Health research programme undertaken by the National Centre for Antimicrobial Stewardship (NCAS) in Australia has combined antimicrobial prescribing surveillance with qualitative research focused on developing antimicrobial use-related situational analyses and scoping AMS implementation options across healthcare settings, including metropolitan hospitals, regional and rural hospitals, aged care homes, general practice clinics and companion animal and agricultural veterinary practices. Qualitative research involving clinicians across these diverse settings in Australia has contributed to improved understanding of contextual factors that influence antimicrobial prescribing, and barriers and facilitators of AMS implementation. This body of research has been underpinned by a commitment to supplementing ‘big data’ on antimicrobial prescribing practices, where available, with knowledge of the sociocultural, technical, environmental and other factors that shape prescribing behaviours. NCAS provided a unique opportunity for exchange and cross-pollination across the human and animal health programme domains. It has facilitated synergistic approaches to AMS research and education, and implementation of resources and stewardship activities. The NCAS programme aimed to synergistically combine quantitative and qualitative approaches to AMS research. In this article, we describe the qualitative findings of the first 5 years.

Judicious use of antimicrobials in human and animal health is a core objective and principle within all national action plans developed to address the global challenge of antimicrobial resistance (AMR). However, there remain significant barriers to achieving this goal, partly because of a paucity of information about how and why antimicrobials are being used, which, if available, could inform efforts to drive improvements. Effective implementation of programmes to improve the quality and safety of use of antimicrobials, i.e. antimicrobial stewardship (AMS), clearly requires both a sociocultural and a technical approach (Figure 1).

**Figure 1.**
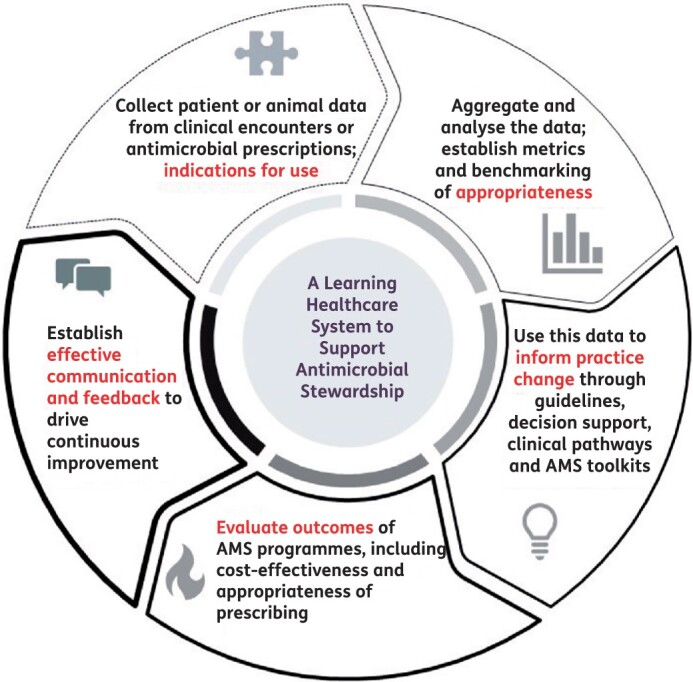
A ‘learning healthcare system’ approach to AMS.

The National Centre for Antimicrobial Stewardship (NCAS) is a One Health research programme focused on monitoring the **quality of antimicrobial use** and the implementation of **antimicrobial stewardship** activities to improve this. The centre has influenced antimicrobial prescribing policy and practice across human and animal health sectors in Australia; it has supported the implementation of Australia’s first ‘National Antimicrobial Resistance Strategy (2015–19)’^1^ and will inform the recently released ‘Australia’s National Antimicrobial Resistance Strategy—2020 and Beyond’.^2^ The programme has aimed to answer the following research questions:

- How are antibiotics being used and what is the appropriateness of use in different settings?
- What are the drivers (prescriber knowledge and attitudes) of antibiotic prescribing?
- What interventions to improve antibiotic prescribing fit workflows and meet the needs of clinicians?

NCAS has facilitated a cross-disciplinary approach to AMS, with experts and research fellows sharing learnings across human and animal health sectors. NCAS researchers have undertaken qualitative research on the knowledge, attitudes and practices of prescribers, consumers and stakeholders in healthcare settings across the One Health continuum. This article aims to provide context-specific information about the challenges and opportunities for AMS in diverse healthcare settings while highlighting setting-specific barriers and facilitators of AMS implementation in Australia as identified through the qualitative research conducted by NCAS. We focus on AMS implementation—describing governance structures, processes and/or surveillance data where relevant—across human and animal healthcare sectors in Australia and highlight the findings of qualitative research undertaken in these settings. The research described demonstrates the value of applying qualitative methods to the study of AMS implementation across the One Health continuum in Australia and of co-locating researchers in one centre to effectively share learnings. Table 1 summarizes the qualitative research projects undertaken by NCAS researchers and collaborators.

**Table 1.** A summary of qualitative research undertaken by NCAS researchers

Study name and authors	Study setting	Qualitative methodology	Participants	Aim	Outcomes/themes	Key message
Qualitative study of the factors impacting antimicrobial stewardship programme delivery in regional and remote hospitals						
Bishop *et al.* 2019^49^	Acute care	Focus groups conducted with a neo-positivist approach and analysed using the framework method^130^	22 participants (8 pharmacists, 6 ID physicians/microbiologists, 3 infection control practitioners/nurses, 3 GPs and 1 clinical nurse administrator)	To describe the contemporary barriers and enablers to AMS programme delivery Australian regional-rural hospitals from the perspective of clinicians with AMS responsibilities	Contextual barriers to AMS include: (i) culture of independence; (ii) self-reliance by local clinicians; (iii) interconnected work-life relationships; (iv) geographical isolation of the hospital influencing antimicrobial choice; (v) lack of understanding of the local context by consulting clinicians (e.g. local resistance)—inability to meaningfully benchmark performance with similar hospitals; and (vi) lack of human resources with ID training. Strategies to support AMS programme delivery in regional-rural hospitals were centrally driven (to provide access to expertise, resources and networking) and locally driven (increased accountability and oversight).	AMS programme delivery in regional-rural hospitals is influenced by factors that are not present in hospitals in major cities, and these must be considered when developing strategies to support regional-rural hospitals to deliver effective AMS programmes.
Sustainability of antimicrobial stewardship programs in Australian rural hospitals: a qualitative study						
Bishop *et al.* 2020^50^	Acute care	Interviews conducted with a neo-positivist approach and analysed using the framework method^130^	15 key informants (5 pharmacists, 4 ID physicians, 2 dual-trained ID physicians and microbiologists, 2 infection control practitioner consultants, 1 microbiologist and 1 GP)	To describe the features of sustained AMS programmes in Australian regional-rural hospitals	The most prominent factors for sustainable AMS programmes in regional-rural hospitals were described as hospital executive support, dedicated AMS resources, network- or area-wide arrangements, passionate champions and adaptability. Challenges to building AMS programmes with these features were identified.	Actions to boost the sustainability of AMS programmes in regional-rural hospitals are required. These include using accreditation as a mechanism to drive direct resource allocation, explicit staffing recommendations for rural hospitals, greater support to develop network arrangements and support to create integrated AMS programmes across acute, aged and primary care.
Influences on surgical antimicrobial prophylaxis decision-making by surgical craft groups, anaesthetists, pharmacists and nurses in public and private hospitals						
Ierano *et al.* 2019^69^	Acute care	Focus groups and interviews; thematic analysis using the COM-B model; TDF^70^ and BCW^72^	14 focus groups; 1 paired interview Health professionals: surgeons, anaesthetists, pharmacists and theatre nurses across 3 public and private acute Victorian hospitals	Primary: To identify barriers and enablers of appropriate SAP prescribing and evidence-based guideline compliance Secondary: To compare the perceptions of health professionals in surgical specialties across both public and private hospital settings regarding these barriers and enablers	SAP prescribing is a complex process that involves multiple professions across the pre-, intra- and post-operative surgical settings. Interventions should aim to increase surgeon engagement, enhance the prioritization of and accountability for SAP, and address the underlying social factors involved in SAP decision-making, such as professional hierarchy and varied perceptions of risk and fears.	The utilization of behaviour-change frameworks to identify barriers and enablers to optimal SAP prescribing supports future development of theory-informed AMS interventions.
Evaluating the implementability of antibiotic surgical prophylaxis guidelines						
Ierano *et al.* 2020^131^	Acute care	GLIA instrument^132^	15 participants were recruited iteratively, with 10 appraisals completed (response rate: 66.7%) that represented all targeted stakeholders; 3 ID physicians, 1 surgeon (5 did not complete the appraisal), 1 infection control consultant, 3 anaesthetists and 2 antimicrobial stewardship pharmacists	To evaluate the implementability of the national guidelines for SAP (TG: A Surgical Prophylaxis chapter)	Guideline recommendations were rated as easily identifiable and concise and were thus facilitators for implementation. The ability to measure guideline adherence and outcomes, and recommendations that were consistent with guideline user abilities and beliefs were also identified as facilitators. Borderline facilitators related to the clarity of the recommendations and whether they were explicit in what to do and in what circumstances. Evidence quality underpinning recommendations (validity), inflexibility of recommendations (flexibility) and the lack of patient data at the point of use (computability) were identified as borderline barriers to implementation. No recommendation reached agreement as being a barrier	The GLIA appraisal demonstrated overall implementability of the current Australian national SAP guidelines. Guideline developers should consider these dimensions to optimize guideline uptake and consequently patient care.
Feasibility and validity of a framework for antimicrobial stewardship in general practice: key stakeholder interviews						
Hawes *et al.* 2020^82^	Primary care	Semi-structured interviews; thematic analysis for feasibility and validity	12 key stakeholders (professionals, not patients)	To determine the feasibility and validity of the proposed AMS framework. A secondary objective was to identify likely bodies responsible for implementation in Australia	The framework was considered valid and feasible. No clear organization was identified to lead AMS implementation in general practice. The current volume-based antibiotic prescription monitoring system was considered insufficient. AMS education for the public, further development of GP education and improved consultation support were strongly recommended. The role of community-based pharmacists and nurses is largely unexplored, but their involvement was recommended.	A clear leader to drive AMS in general practice is essential for an action framework to gain traction. Strategies to monitor and provide feedback on antibiotic prescribing need to consider prescribing appropriateness and patient outcomes.
How do general practitioners access guidelines and utilise electronic medical records to make clinical decisions on antibiotic use? Results from an Australian qualitative study						
Biezen *et al.* 2019^83^	Primary care	Exploratory qualitative study using focus groups and thematic analysis with the theory of planned behaviour framework^133^	26 GPs from 5 general practices	To explore how GPs access and use both guidelines and EMRs to assist in clinical decision-making when prescribing antibiotics in Australia	GPs expressed that current EMR systems do not provide CDS to assist with antibiotic prescribing. Younger and less experienced GPs were more likely to access guidelines than more clinically experienced GPs. A lack of access to guidelines and perceived patients’ expectation and demand for antibiotics were barriers to guideline-concordant prescribing. However, guidelines that were easy to access and navigate, free and embedded within EMRs, and fitted into the clinical workflow were seen as likely to enhance guideline use. Barriers to the use of antibiotic guidelines include GPs’ experience, patient factors, practice culture, difficulty of access and perceived cost of guidelines.	Guidelines should be made available, accessible and easy to use, with minimal cost to practising GPs to reduce inappropriate antibiotic prescribing and to promote more rational use of antibiotic in the community.
Dissonant views—GPs’ and parents’ perspectives on antibiotic prescribing for young children with respiratory tract infections						
Biezen *et al.* 2019^84^	Primary care	Mixed-methods study with semi-structured in-depth interviews and a short questionnaire	Interviews with 20 GPs and focus groups with 50 parents and carers of children under 5 years of age	To explore GPs’ and parents’ perceptions regarding antibiotic prescribing for RTIs in young children	GPs believed that parents expect antibiotics for RTIs and that they would go elsewhere, and hence were more likely to prescribe antibiotics if parents were insistent. GPs suggested that there would be less conflict if parents were better educated on appropriate antibiotic use. In contrast, parents demonstrated good knowledge of RTIs and appropriate antibiotic use. Their main expectation from GPs was to obtain a diagnosis, discuss management and receive reassurance that the illness was not serious. Parental satisfaction with GPs was not dependent on receiving antibiotics, and they would not seek another GP if antibiotics were not prescribed. GPs and parents have dissonant views on antibiotic prescribing for RTI in young children. GPs perceived parents wanting a diagnosis and reassurance as contributing to pressure to prescribe antibiotics.	Targeted training for both GPs and parents to improve communication and reassurance that satisfaction is not related to receiving antibiotics may reduce unnecessary antibiotic prescribing for RTI in young children may overcome these barriers.
Developing a clinical decision support tool for appropriate antibiotic prescribing in Australian general practice: a simulation study						
Manski-Nankervis *et al.* 2020^85^	Primary care	An interpretive descriptive approach using a co-design methodology, followed by evaluation with 2 case scenarios conducted in a simulated environment	8 GPs participated in 2 simulated consultations	To explore the use, acceptability, and feasibility of a CDS tool that incorporates evidence-based guidelines and consumer information that integrates with the EMR	GPs thought the consultations were ‘real’ and representative of real-life consultations; 7 of 8 GPs were satisfied with the usability of the tool. Key findings included that the tool assisted with clinical decision-making and informed appropriate antibiotic prescribing. Key factors such as accessibility and ease of use, quality of content, layout and format determined whether GPs said that they would access the tool in everyday practice. Integration of the tool at multiple sites within the EMR facilitated access to guidelines and assisted in ensuring that the tool fit the clinical workflow. Piloting of the tool in general practices to assess the impact and feasibility of use in real-world consultations.	The CDS tool that integrated evidence-based guidelines and consumer information into the EMR was acceptable to GPs.
Shared decision support in general practice: an antimicrobial stewardship strategy to promote appropriate use of antibiotic in primary care						
Biezen *et al.* 2021 (government report)^a^	Primary care	An interpretive descriptive approach using a co-design methodology, followed by semi-structured in-depth interviews and thematic analysis	The study comprised three components: (i) a literature review to assess current decision support tools for antibiotic prescribing for RTIs, SSTIs and UTIs; (ii) co-design with 5 healthcare providers and 6 consumers to develop patient information tools; and (iii) piloting of patient information tools in 8 metropolitan and regional general practices in Victoria to assess user acceptance	To develop and evaluate robust decision support tools in the form of patient information sheets to assist both healthcare providers and patients to guide antibiotic use in primary care	Both healthcare providers and consumers/patients emphasized the importance of simple, concise and inclusive language, with design and formatting to engage users and to improve usability in the patient information sheets. While the use of the patient information sheets was lower than anticipated due to the impact of COVID-19, GPs used the information sheets where possible during the intervention period and both healthcare providers and consumers thought they were acceptable and easy to use. The patient information sheets provided GPs an alternative to prescribe antibiotics, reinforced their treatment and management options, and increased patient knowledge around disease conditions and treatment and management options.	The study demonstrated high usability and acceptance of the seven patient information sheets for common infections. Co-designing with healthcare providers and consumers provided a robust methodology to ensure the product met the needs of end-users.
Evaluating the implementation of a pilot quality improvement program to support appropriate antimicrobial prescribing in general practice						
Biezen *et al.* 2021^b^	Primary care	Implementation of a pilot programme using qualitative approach with thematic analysis interpreted using the CP-FIT^134^	31 GPs participated in the programme, with 11 GPs and 3 practice managers participating in follow-up focus groups and interviews to explore the acceptability and feasibility of the programme	To evaluate the implementation of a quality improvement programme (Guidance GP) in 3 general practices in Melbourne, Australia, between November 2019 and August 2020	The quality improvement activities were acceptable to GPs, if they accurately fit GPs’ decision-making process and workflow. Providing clinically meaningful information in the form of audit and feedback to GPs was also seen as important. However, barriers identified included time needed to coordinate the programme and costs to implement the programme. Some facilitators identified included a ‘whole of practice’ approach.	The study established that implementing a QI programme will need to consider barriers such as cost to the practice, programme fitting into the GP workflow and data accuracy. However, facilitators such as an enthusiastic practice-wide approach, positive practice champions, and the passion to reduce inappropriate antibiotic prescribing can contribute to the success of the programme.
Divergent and convergent attitudes and views of general practitioners and community pharmacists to collaboratively implement antimicrobial stewardship programs in Australia: a nation-wide study						
Saha *et al.* 2021^90^	Primary care	SEIPS 2.0 Model^135^ guided by a human factor engineering approach	999 participants; quantitative responses: 386 GPs and 613 CPs; qualitative responses: 221 GPs and 592 CPs	To explore the convergent and divergent attitudes of GPs and CPs about AMS implementation and to identify challenges of GP–CP collaboration in AMS	CPs’ need for AMS training was significantly higher than that of GPs. GPs used TG: A at a much higher rate than CPs. There was no interprofessional difference in using patient information leaflets and point-of-care tests. Though CPs were more willing to collaborate than GPs, both believed that policies supporting GP–CP collaboration are required to implement AMS strategies. Challenges of collaboration in AMS were found at personal, logistical, organizational and policy levels.	There are opportunities to implement AMS through collaboration between GPs and CPs in primary care, but a collaborative system structure and GP-pharmacy practice agreements are key to improving interprofessional trust, competencies and communications for AMS.
Antimicrobial stewardship near the end of life in aged care homes						
Dowson *et al.* 2020^105^	ACHs	Interviews and thematic analysis using the BCW	12 nurses, 6 GPs, 2 pharmacists providing care to ACH residents	To explore how ACH health professionals perceive antimicrobial use and potential antimicrobial stewardship activities near the EoL in ACHs	Two major themes emerged: (i) accreditation standards provide motivation for behaviour change and AMS activities near the end of life in ACHs should clearly be part of an ACH nurse’s role; and (ii) AMS activities near the EoL in ACHs must address family confidence about resident wellbeing and should be inclusive of family involvement.	Accreditation standards are key motivators for behaviour change in ACHs. Messaging should highlight that AMS improves care.
The role of nurses in antimicrobial stewardship near the end of life in aged-care homes: a qualitative study						
Dowson *et al.* 2020^106^	Aged care	Interviews and thematic analysis using the COM-B model and TDF	20 healthcare professionals providing routine care in ACHs	To investigate the potential opportunities for nurses to undertake antimicrobial stewardship activities near the EoL in ACHs.	ACH nurses are influential in antimicrobial decisions near the EoL in routine care. Nurses are leaders in advance care planning, care coordination and care provision in environments with stopgap and visiting medical resources, and have social influence among residents, families and medical professionals during critical conversations near the EoL. ‘Fear-based’ social influences on antimicrobial prescribing can emerge if there have been past negative social interactions between nurses and families in the aged care environment.	There are opportunities for ACH nurses to undertake AMS activities near the EoL in the delivery of routine care.
Appraisal of the Australian Veterinary Prescribing Guidelines for antimicrobial prophylaxis for surgery in dogs and cats						
Hardefeldt *et al.* 2019^123^	Veterinary medicine	GLIA and AGREE II^136^ instruments	GLIA: 2 small animal specialist surgeons AGREE II: 12 veterinarians, including 2 specialist surgeons, 4 small animal specialists with an interest in AMS and 6 GP veterinarians	To determine the validity and implementability of Australian SAP guidelines	GLIA: The specialist surgeons either agreed or strongly agreed that the guidelines were executable, decidable, valid and novel, and but were neutral on flexibility and measurability. AGREE: The veterinarians were satisfied with the scope and purpose of the guidelines, stakeholder involvement, rigour of development, clarity of presentation, applicability and editorial independence, resulting in a global scaled score of 76%.	The Australian Veterinary Prescribing Guidelines for SAP are considered valid and implementable.
Barriers to and enablers of implementing antimicrobial stewardship programs in veterinary practices						
Hardefeldt *et al.* 2018^121^	Veterinary medicine	Mixed-methods approach using online questionnaire followed by group interviews and thematic analysis	Survey of 184 veterinarians followed by 13 group interviews with 39 veterinarians from companion animal, equine and bovine practice	To investigate three key areas: attitudes to and experiences of AMR, current AMS processes, and needs for and barriers to proposed components of AMS programmes	Veterinarians were concerned about AMR; however, there were major barriers to improving antibiotic prescribing, including a lack of independent guidelines, lack of access to education and training, client expectations of antibiotics and high cost of culture and susceptibility testing.	Veterinary participants were generally motivated to improve their antibiotic use, and some of the barriers to optimal prescribing can be overcome with appropriate prescribing guidelines and training.
In-water antibiotic dosing practices on pig farms						
Little *et al.* 2021^115^	Veterinary medicine	Mixed-methods approach using detailed online questionnaire followed by semi-structured in-depth interviews and thematic analysis	Survey of 25 Australian pig farmers, followed by one-on-one interviews with each	To investigate how in-water antibiotics are administered on Australian pig farms to identify areas for improvement	Large variations were found in the way in-water antibiotics were used across pig farms. In particular, the type of antibiotics given, the equipment used to deliver the antibiotics, the way that doses are calculated and prepared as solutions, and the timing and frequency of doses. These variations are likely to have significant impact on the effective dose received by each animal.	Significant opportunities exist to reduce overall antibiotic use on pig farms by increasing the effectiveness of in-water dosing. Farm managers should be provided with measurement systems, technical guidelines, and training programmes to optimize their antibiotic use.

AGREE II, appraisal of guidelines for research and evaluation version 2; BCW, behaviour change wheel; CP, community pharmacist; CP-FIT, clinical performance feedback intervention theory; GLIA, guideline implementability appraisal; ID, infectious diseases; RTI, respiratory tract infection; SSTI, skin and soft tissue infection.

a R. Biezen, J. Manski-Nankervis, S. Ciavarella, T. Monaghan, K. Somasundaram, R. Ingram and K. Buising, unpublished data.

b R. Biezen, K. Buising, T. Monaghan, R. Ball, K. Thursky, R. Cheah, M. Clark and J. Manski-Nankervis, unpublished data.

## The impact of the Australian National Antimicrobial Prescribing Survey (NAPS)

Initially developed by Thursky, Buising and James^3^ in a research project, the NAPS has been adopted as a core instrument to support AMS programmes in hospitals and residential aged care homes, and to provide data for the national antimicrobial use and AMR surveillance programme (Antimicrobial Use and Resistance in Australia [AURA]).^4^ Using a ‘plan, do, study and act’ (PDSA) improvement cycle,^5^ the platform has undergone continuous improvement since 2013, and now comprises four modules: the Hospital NAPS,^6–12^ Surgical NAPS,^13–15^ Aged Care NAPS^16–19^ and Quality Improvement NAPS. Despite the voluntary nature of the survey activities (point-prevalence, cohorts or directed surveys), participation has continued to increase across both public and private institutions.^6–12^ Unique internationally, the NAPS platform has demonstrated the feasibility and acceptability of measuring the appropriateness of antimicrobials being used, rather than a limited assessment of guideline compliance, and collecting data on all antimicrobials rather than just a few nominated drugs.

Qualitative research has informed the expansion of the NAPS. Recently, the need for a dedicated, detailed survey of hospital antifungal use was identified.^20^ In 2020, Khanina *et al.*^21^ undertook an international Delphi study with 82 experts in antifungal use from 17 different countries; participating experts achieved consensus on 38 antifungal stewardship metrics, including antifungal consumption, quality of antifungal prescribing and management of invasive fungal infection, and clinical outcomes.^21^ Many of the existing NAPS appropriateness metrics were deemed to have moderate to high feasibility for routine collection, and this information will guide the design of a dedicated module.^21^ Similarly, the Hospital NAPS has been used in several international settings, including Canada, New Zealand, the United Kingdom, Bhutan, Malaysia^22^ and Fiji, and feedback from the clinical leaders at these sites has been collected via surveys and key informant interviews to iteratively improve the tool to meet needs.

## Acute care (hospitals)

The introduction of AMS as a dedicated hospital accreditation standard in Australia in 2011^23^ (updated in 2015) was a major driver for implementation of funded AMS programmes in hospitals. The standard explicitly supports the need to provide access to national antimicrobial prescribing guidelines, to use antimicrobial restriction and approval processes, and to monitor consumption and *appropriateness* of antimicrobial use.^24^ Australian researchers have made major contributions to the AMS literature focused on antimicrobial use in hospitals; these studies have described antimicrobial use in intensive care,^25–28^ emergency departments^29^^,^^30^ and paediatrics;^31^^,^^32^ sepsis pathways;^33^^,^^34^ antimicrobial-allergy de-labelling programs;^35–37^ and antifungal stewardship.^38^ Independent, expert-led antimicrobial prescribing guidelines, ‘Therapeutic Guidelines: Antibiotic’ (TG: A), have been available in Australia since 1978, enabling standardization of evidence-based prescribing and prescribing quality audits.^24^^,^^39^ NCAS researchers made major contributions to providing an evidence base for computerized clinical decision-support (CDS) systems, particularly antimicrobial approval systems for AMS, with internal and external peer-reviewed evaluations of tools developed by NCAS researchers.^26^^,^^40–43^ In the last 5 years, the implementation of electronic medical records (EMRs) has increased in some Australian hospitals and facilitated AMS programmes by enabling efficient antimicrobial usage auditing and linkages to computerized CDS systems.^26^^,^^27^^,^^41^^,^^44^^,^^45^

### Challenges in regional and rural Australia

Regional and rural hospitals in Australia have context-specific needs and challenges relating to AMS,^46^ and there are disparities in AMS implementation, reflecting broader differences in healthcare delivery between metropolitan and regional and rural settings.^47^ Analysis of data from the Hospital NAPS suggests that compared with major-city hospitals, regional and rural (‘rural’) hospitals have higher levels of inappropriate antimicrobial prescribing for particular antimicrobials (e.g. ceftriaxone) and common infections (e.g. cellulitis and sepsis).^48^ Two qualitative studies by NCAS researchers Bishop *et al.*^49^^,^^50^ explored challenges and opportunities for AMS in rural health services. The first study included focus groups with health professionals involved in AMS programmes in their health service.^49^ Broadly, barriers to the implementation of AMS programmes in rural settings include competing demands for resources; difficulty in recruiting staff; lack of training and education; limited resources for information technology; limited pharmacy resources; distance (resulting in isolation from the larger centres); and lack of support from some medical professionals.^49^ These findings build on other Australian work in rural settings.^46^^,^^51–54^ Unique contextual barriers include a culture of independence and self-reliance by local clinicians; interconnected work-life relationships; geographical isolation of the hospital influencing antimicrobial choice; lack of understanding of the local context (e.g. local AMR patterns); an inability to meaningfully benchmark performance with similar hospitals; and lack of professionals with infectious diseases training.^49^ Interviews with key opinion leaders and innovators in rural AMS helped identify that the most prominent factors for sustainable AMS programmes in rural hospitals include hospital executive support, dedicated AMS resources, network or area-wide arrangements, passionate AMS champions and adaptability.^50^ These findings generated key recommendations to boost the sustainability of rural hospital AMS programmes: using accreditation as a mechanism to drive direct resource allocation; defining AMS staffing recommendations for rural health services; supporting more AMS network arrangements involving rural health services; and integrating rural AMS programmes across acute, aged and primary care.^50^ This qualitative work, combined with the analysis of the NAPS data, led to the conceptualization of a cellulitis management quality improvement study.^55^ Bishop *et al.*^55^ developed a bundle of care (operationalized into a cellulitis management plan) aimed at increasing the appropriateness of antibiotic therapy for cellulitis. Drawing on findings about the value of networks in achieving AMS outcomes, this project involved collaboration among three regional and rural health services and was evaluated using the RE-AIM framework.^56^

Qualitative research has highlighted that rural hospitals have developed strategies to augment AMS implementation in the face of existing challenges and resource gaps.^50^ These strategies include: use of centralized (‘hub and spoke’) models, where tertiary or principal referral hospitals provide network-wide AMS support and access to infectious diseases expertise;^41^^,^^57^ use of computerized decision-support systems for AMS across networks of regional hospitals;^41^^,^^58^ visits by infectious diseases specialists to smaller hospitals; and use of telehealth.^57^^,^^59^^,^^60^ AMS programmes that are led by non-infectious diseases doctors, pharmacists, infection control practitioners and nurses are now common in Australian rural hospitals.^49^^,^^50^^,^^61^^,^^62^ Support for these staff is being bolstered through AMS network arrangements. There are opportunities for new partnerships to be created, particularly with primary care and aged care, given their interconnectedness with acute hospital care in rural settings.^63^

### An example of an AMS challenge in acute care: surgical antimicrobial prophylaxis (SAP)

SAP is the most common indication for antibiotic use in Australian hospitals, with high rates of inappropriate prescribing.^6–12^ Activities are being developed and promoted to improve SAP,^64^^,^^65^ including widespread participation in the Surgical NAPS.^13–15^ The Surgical NAPS data reveal variation in the quality of SAP prescribing practices in Australia^66^^,^^67^—in both peri-operative (timing, choice) and post-operative prescribing (duration)—across all surgical specialties, with poor overall appropriateness of antibiotic use (48.7%).^67^ AMS interventions will need to be tailored to specific specialties, addressing systemic, behavioural and environmental factors in each.^68^ In Australia, there is no standardized and/or national approach for routine monitoring of surgical site infections to facilitate benchmarking and quality improvement. Additionally, there are no current efforts to link administrative and surveillance datasets to monitor the impact of improvement programmes. Qualitative research suggests these elements may collectively serve as a significant driver for prescriber behaviour change.^69^

NCAS researchers Ierano *et al.*^69^ undertook qualitative work to examine decision-making processes related to SAP prescribing and guideline compliance. Their study involved focus groups and interviews with key stakeholders across the peri-operative pathway: surgeons, anaesthetists, peri-operative nurses and clinical pharmacists, with analysis guided by the theoretical domains framework (TDF)^70^ and the capabilities, opportunities, motivators-behaviour (COM-B) model.^71^^,^^72^ Six key themes relating to clinicians’ perceptions about decision-making for antimicrobial use across the surgical setting were identified: SAP prescribing skills are considered a low priority; prescriber autonomy takes precedence over guideline compliance; the social codes of prescribing reinforce established practices; there is a need for improved communication, documentation and collection of data for action; fears and perceptions about risk hinder appropriate SAP prescribing; and there is a lack of clarity regarding roles and accountability for SAP prescribing.^69^ This research generated recommendations for SAP improvement. To facilitate appropriate SAP prescribing, there is a need to support prescribing quality data benchmarking, and to develop the ability to link appropriateness of SAP use to patient outcomes (such as surgical site infections, readmission, mortality, sepsis and *Clostridioides difficile* infection). Opportunities to capitalize on existing workflows*,* such as the ‘time-out’ process^73^ and the enhanced recovery after surgery (ERAS) protocol,^74^ to support SAP prescribing were identified. Cultural barriers to AMS for SAP prescribing, such as the influence of professional hierarchy, and fears and risk perception, can be addressed through leadership engagement and evidence-based reinforcement of information on patient safety and quality of care.

## Primary care

Primary care is where most of the antibiotic prescribing for humans occurs in Australia, and data do suggest that there is likely to be an opportunity for AMS in this sector. The Australian community uses nearly twice the average volume of antibiotics per capita of Organisation for Economic Co-operation and Development (OECD) countries.^75^^,^^76^ The frequency of antibiotic prescribing per patient presentation in general practice varies between 5% and 15%.^77^ The types of antibiotics being prescribed by Australian general practitioners (GPs) appear to be of broader spectrum than those prescribed by their peers in other countries,^4^ with amoxicillin/clavulanic acid and cefalexin among the most commonly prescribed antibiotics.^4^ The patterns of antibiotic use do not appear to align well with national guidelines, especially for several respiratory tract indications (including bronchitis, pharyngitis and otitis media).^78–80^

NCAS researchers Hawes *et al.*^81^ conducted a literature review to identify a possible framework for AMS in general practice, which identified six key components: governance; monitoring of antibiotic prescribing and AMR with feedback to GPs; education of the public and health professionals about AMR and AMS; consultation support; pharmacy- and nursing-based approaches; and research. They interviewed key Australian stakeholders to determine the feasibility and validity of the framework.^82^ These stakeholders identified that there was no clear leadership for AMS in Australian general practice, and that a focus on prescribing appropriateness and patient outcomes in antibiotic prescribing audit and feedback strategies would be useful.^82^ Stakeholders agreed that community education (targeting the general public) was necessary to support general practice AMS, and that while community pharmacists may require support to be involved, having access to non-dispensing pharmacists (also referred to as clinical pharmacists or practice pharmacists) in general practice may be useful.^82^ Electronic decision-support for GPs was also strongly supported.^82^

Biezen *et al.*^83^ undertook qualitative research on GPs’ use of both prescribing guidelines and EMRs for clinical decision-making when prescribing antibiotics in Australian primary care clinics. This research highlighted both structural factors (such as a lack of integration between prescribing guidelines and EMRs) and sociocultural factors (such as clinician preference and practice culture relating to guideline use and evidence-based prescribing, and patient expectations for antibiotic prescribing) that influence guideline uptake and conformity.^83^ Previous research by Biezen *et al.*^84^ indicated a dissonance between GPs’ perceptions about patient demands for antibiotic prescribing for upper respiratory tract infections in children and patients’ self-reported expectations.

In collaboration with the University of Melbourne’s Department of General Practice, NCAS researchers developed a CDS tool that incorporated evidence-based guidelines (TG: A) into the EMR. This was tested with GPs in simulated consultations and assessed qualitatively.^85^ In addition to a pilot audit and feedback programme, the General Practice NAPS, which provided feedback to GPs in the form of a report and educational webinar, a quality improvement programme called Guidance GP was developed through this programme of work. The latter programme consisted of an embedded audit tool that extracted data when an antibiotic was prescribed and prompted GPs to enter an indication for prescribing if not recorded in the EMR; a written feedback report containing information on prescribing volume, compliance with guidelines and appropriateness; and educational webinars and in-practice quality improvement support. The researchers piloted and evaluated the programme qualitatively in several clinics and found that the quality improvement activities were acceptable to GPs and fitted into their decision-making process and workflow.^85^

GPs’ perceptions about AMS have been assessed by Saha *et al.*^86^ through a nationwide survey. Of 389 GP respondents, 68.9% were familiar with AMS; 83.2% referred to TG: A and 72.2% used delayed prescribing as an AMS strategy.^86^ However, only 18.4% used point-of-care tests, 20.2% used patient information leaflets and 9.8% used audit and feedback strategies.^86^ The participating GPs were receptive to AMS training, integration of guidelines with EMR and policies limiting the prescribing of selected antimicrobials.^86^ GPs’ perceptions about the potential for community pharmacists to contribute to AMS were mixed: 50.5% and 63% were receptive to community pharmacists’ recommendations on antimicrobial choice and dose, respectively, and 60% supported fostering greater GP–community pharmacist collaboration.^86^ A nationwide survey of community pharmacists by Saha *et al.*,^87^ with 613 participants, found that 73% were familiar with AMS and that 76.5% felt that they would require specialized training. Community pharmacists reported that they counselled patients (97%) and reviewed drug interactions or allergies (93.8%) often but less commonly referred to prescribing guidelines (45.5%) or assessed the guideline-compliance of prescribed antimicrobials (37.9%).^87^ The participants perceived that GPs were not receptive to interventions about antimicrobial choice (82.6%) and dosage (68.6%).^87^ Our work has scoped the potential for collaborative AMS initiatives in primary care that may capitalize on the convergence of GPs’ and community pharmacists’ perceptions about AMS opportunities and facilitators.^88–92^ Though there were differences in GPs’ and community pharmacists’ receptiveness to participation in collaborative GP–community pharmacist group meetings (54.9% versus 82.5%) and antimicrobial audits (46.1% versus 86.5%), opportunities to improve interprofessional trust, technological capabilities and organizational and environmental factors were identified.^90^

Interventions in primary care by other Australian research groups, ranging from public education to more specific prescriber training and education,^93^^,^^94^ have been trialled in Australia, but much more needs to be done.^95^

## Aged care

One in seven Australians is over 65 years of age,^96^ and the demand for aged care homes (ACHs) and in-home care continues to increase.^97^ AMS programmes have not been widely implemented in ACHs but will be driven by accreditation standards that were introduced in 2019.^98^ Importantly, consecutive annual Aged Care NAPS surveys have highlighted several targets for action.^16–19^ For example, the 2019 report showed that the proportion of residents who had signs and/or symptoms of at least one suspected infection on the survey day was 3.1%, whereas the proportion of residents prescribed at least one antimicrobial was 8.2%.^19^ Prolonged antibiotic use for urinary tract infection (UTI) prophylaxis was widespread, as was the use of topical antimicrobials. Furthermore, 24.5% of prescriptions did not have a documented indication for prescribing, and 35.3% did not have either a review date or a stop date.^19^

Management of infections requiring antimicrobial therapy may be difficult in ACHs due to atypical clinical presentations in older people, the inability of residents to communicate symptoms due to cognitive impairment and poor availability of diagnostic tests.^99^ NCAS researchers identified that these challenges, coupled with the desire to treat suspected infections ‘just in case’ residents get sicker (often to avoid hospital transfer), and possibly perceptions of the increased risk posed by drug-resistant infections, can lead to over-prescribing.^100^^,^^101^ Additionally, some antibiotic prescriptions may be commenced empirically but not reviewed in a timely manner; thus, prolonged courses of therapy are, reportedly, common.^102^^,^^103^ It was reported that poor interpretation of microbiology results could also potentially lead to over-treatment of colonizing bacteria, such as in the treatment of asymptomatic bacteriuria, non-infected skin ulcers, or colonizing bacteria in sputum samples.^104^ Strategies to improve AMS in ACHs, including the development of simple clinical pathways and guidelines for the diagnosis and management of urinary sepsis, respiratory tract infection and skin and soft tissue infection, have been suggested. The qualitative findings also suggest that strategies to ensure appropriate documentation of indications and plans for antibiotics (including undertaking timely review at 48–72 h) and to limit antibiotic duration are needed, as are more precise criteria to help govern when to send samples for microbiological testing and assistance with interpreting the significance of any bacteria isolated from such specimens. This will inform next steps in aged care AMS.

Residents in the final month of life are increasingly likely to be prescribed an antimicrobial, commonly without having signs and symptoms of infection. Dowson *et al.*^105^^,^^106^ described the perspectives of health professionals on antimicrobial use near the end of life (EoL) in ACHs and investigated the potential opportunities for AMS activities. Interviews were conducted with nurses, GPs and pharmacists with a diversity of years of experience in providing routine care to residents of ACHs in Victoria in different facility locations (metropolitan and rural) and types (public and private). Reported workflow-based challenges in ACHs included limited on-site staff resources and the use of multiple off-site care providers.^106^ Opportunities for ACH nurses to undertake AMS activities near the EoL in the provision of routine care were identified. Support for ACH nurses to make decisions substantiated by evidence-based clinical teaching and through improved care coordination relating to infection management was highlighted as a potential facilitator of AMS involvement. The importance of AMS activities near the EoL also addressing family confidence about resident wellbeing was identified. Prior discussion about the role of antimicrobial use in EoL care was thought to be relevant,^105^ as was discussion about the role of non-pharmacological therapies.

## Veterinary care

Use of antimicrobials in veterinary medicine is essential to ensuring animal health and welfare, and the security and safety of food. However, there is ample scope for the introduction of AMS across all areas of veterinary medicine to optimize appropriate antimicrobial usage in Australia. In Australia, there are publicly available data on the volumes of antimicrobials sold in the animal health sectors, but detailed species-level data and surveillance of end use and appropriateness of use are lacking. These gaps in our understanding have been partially addressed by recent studies in some areas of veterinary practice.^107–112^

Use of antimicrobials in food-producing animals is strictly regulated in Australia. Most veterinary antimicrobials are available only by prescription and their use by veterinarians is regulated by state legislation and labelling restraints. Antimicrobial use is monitored through chemical residue testing of food of animal origin. The Australian Pesticides and Veterinary Medicines Authority (APVMA) periodically reports on the total quantity of antimicrobial products imported (in tonnes of active constituents) for animal use in Australia. The most recent data showed that 98% of veterinary antimicrobials sold in 2005–10 were used for food animals.^113^ Over half of the antimicrobials were coccidiostats, most of which are used to prevent coccidiosis in chickens. These antimicrobials are not used in humans and are thus not considered to pose a risk in terms of development of AMR of concern to human health. Macrolides and tetracyclines were the antibiotic classes used in the greatest volumes, according to the APVMA data. In a survey conducted by Crabb *et al.*,^110^ Australian poultry veterinarians also reported using amoxicillin for some common diseases.

Antimicrobials administered to production animals are mainly regarded to be of low importance in the Australian rating system, and antimicrobials of high human medical importance have very limited use in animal production in Australia; only one late-generation cephalosporin and one streptogramin antibiotic are registered for food-producing animals: ceftiofur, for use in the treatment of respiratory tract infections in cattle, and virginiamycin. In contrast to many other countries, the use of fluoroquinolones has never been permitted in food production animals in Australia. However, there is room for improvement. NCAS research has identified suboptimal antimicrobial dosing and inappropriate timing to be common in SAP in cattle,^112^ horses,^111^ dogs and cats,^107^ just as in humans.

Improving the delivery of antibiotics to production animals is an important focus of AMS globally. The vast majority of antimicrobials used in production animals are provided in feed or drinking water in intensive livestock industries (e.g. pigs, chickens and feedlot cattle) as it is the only practicable way to treat a large number of individuals simultaneously. However, the effectiveness of in-water delivery had not been investigated previously.^114^ Little *et al.*^115^ undertook a mixed-methods study with pig farm managers, which demonstrated a great deal of variation in the way that antimicrobial regimens are calculated, prepared and delivered in drinking water, leading to major differences in dosing and wastage. Farm managers lacked a full appreciation of the complexities involved in ensuring each animal receives an appropriate antimicrobial dose for an appropriate period. This study identified important opportunities for optimizing antimicrobial use in pig farming, by providing farm managers with guidelines for in-water medication and technical training on in-water antimicrobial use, as well as implementing on-farm monitoring of antimicrobial use.

Australia has one of the highest levels of pet ownership in the world. Companion animals, including horses, receive more intensive veterinary care (and hence are more likely to be treated with a wider range of antimicrobial drugs) than animals in agricultural production. Companion animal veterinarians predominately use β-lactam antibiotics in both medical^108^ and surgical scenarios,^107^^,^^111^ although use of third-generation cephalosporins and fluoroquinolones is common in some medical scenarios in dogs and cats.^108^

Under-dosing,^116^^,^^117^ inappropriate timing of administration in relation to surgical incision and excessive durations of therapy in dogs, cats and horses are common issues.^107^^,^^111^ Problems with labelling legislation, resulting in the persistence of historical and incorrect dosing regimens on the labels of older antimicrobials, appear to be contributing to under-dosing in some instances.^111^^,^^118^ The use of novel data sources such as medication records in insurance databases and techniques such as natural language processing by NCAS researchers has enabled insights into population-level patterns of antimicrobial use in companion animals in Australia.^109^^,^^119^

Understanding local AMR patterns and trends is an important cornerstone of AMS efforts. Unfortunately, pooled AMR data are not widely available for animals in Australia. However, our recent research shows that companion animal urinary pathogens in Australia remain reassuringly susceptible to low-importance antimicrobials such as trimethoprim/sulfonamides and amoxicillin,^120^ and these antimicrobials are recommended in prescribing guidelines. Despite this, Australian veterinarians are much more likely to treat UTIs with medium-importance amoxicillin/clavulanic acid or high-importance cefovecin.^109^ The veterinary team of NCAS is undertaking in-depth qualitative research to explore the complex reasons behind such guideline non-compliance and excessive-spectrum antimicrobial use by companion animal veterinarians. This work will build on previous qualitative research by Hardefeldt *et al.*^121^ with companion animal, equine and bovine veterinarians, which found that major barriers to AMS implementation include the high cost of veterinary microbiological testing, client expectations about antibiotics, poor access to continuing veterinary education and training and a lack of industry-independent veterinary guidelines for antimicrobial use.

This work also showed that Australian veterinarians are concerned about the animal and human health consequences of their prescribing, take pride in providing high-quality veterinary services and are willing to change practice.^121^ Our group has since developed and validated independent and transparent antimicrobial prescribing guidelines for veterinarians.^122^^,^^123^ The cost of diagnostic microbiology continues to inhibit the widespread use of culture and susceptibility testing in veterinary practices in Australia. This probably contributes to overuse of antimicrobials, as veterinarians treat suspected infections ‘just in case’ the animal deteriorates.^124^ Cheaper and more rapid diagnostic tests are urgently needed.

Development of a national dataset of veterinary antimicrobial consumption is another key aim, although there are many barriers to achieving this. A limitation of the APVMA data is that each antimicrobial is recorded against the animal species in which its use is registered, rather than the species in which it is ultimately used. Off-label prescribing rights allow the use of a medicine to treat an animal in a way that is not described on the registered label where that use is not specifically prohibited by the label. Off-label use of antibiotics in food animals may occur, but it is largely limited by restrictions on the maximum permitted residue levels in food products. Greater clarity about the actual end usage of antimicrobials in production animals would be of considerable assistance in developing strategies to optimize antimicrobial use and directing efforts to identify alternative animal health strategies.

The key contributor to limiting antimicrobial use in veterinary medicine in Australia has been our long and largely successful history of national biosecurity, which has ensured our freedom from many major infectious diseases and has also limited pathways for introduction of multidrug-resistant organisms from other countries. This has been complemented by the implementation of farm-level biosecurity measures, with farm biosecurity plans increasingly becoming a component of farm quality assurance programmes. In the poultry industries, the development and introduction of comprehensive vaccination programmes has controlled much of the burden of infectious disease, but the limited availability of effective vaccines for a number of major bacterial diseases of pigs necessitates continued antimicrobial therapy.

Implementation of AMS programmes in veterinary practices in Australia is in its infancy.^109^ Many of the building blocks for AMS—such as methods for monitoring antimicrobial use at a local level and for auditing and reviewing antimicrobial prescribing, and protocols and templates for antimicrobial stewardship policies—have not yet been adopted widely in veterinary practice, and a lack of financial incentives for veterinarians working in private businesses to undertake the significant work of AMS is a major challenge. Education of veterinarians about AMS and assistance in developing AMS programmes are critical.^121^^,^^124^ A joint project between the Australian veterinary schools and the Commonwealth government to develop a training programme for veterinarians working in clinical practice may help to address this.^125^ Qualitative projects are also underway in NCAS to understand non-clinical influences on Australian companion animal veterinarians’ prescribing decisions, and the lessons learned from an implementation trial of an AMS programme across 135 companion animal veterinary practices. An international veterinary Delphi study is being undertaken to reach consensus on the role of highest-priority critically important antimicrobials in veterinary medicine.

These studies highlight the importance of understanding structural, cultural, behavioural and technological barriers that affect implementation of AMS programmes. A striking outcome has been the ability for researchers in different sectors to share learnings and leverage observations. The next step is to employ a ‘learning health systems’ approach, utilizing health services research and implementation science methodologies (Figure  1) to act on these findings.

There is an imperative to build research workforce awareness and capacity in qualitative research to address AMR. A consensus paper from the Joint Programming Initiative on Antimicrobial Resistance (JPIAMR) Working Group on behavioural approaches to implementing AMS programmes outlined key research priority areas.^126^ The authors provide guidance for developing research proposals that incorporate metrics, outcomes of AMS intervention studies, the use of the StaRI framework for implementation studies,^127^ and behaviour change interventions based on theoretical frameworks.^128^ A more recent review of current evidence and international engagement with stakeholders from healthcare, public health, research, patient advocacy and policy reinforced the importance of context, culture and behaviours as a major research priority.^129^

## Conclusions

Implementation of AMS programmes still constitutes a major challenge in regional and rural hospitals, primary care, aged care and animal health in Australia, despite the high quality of our healthcare systems. Recognition of accreditation programmes as a driver for AMS and the availability of the comprehensive NAPS programme have facilitated AMS efforts in some areas, with research providing unique insights into key targets for action. The qualitative research performed by the NCAS team has contributed to substantive gains in information required to guide AMS interventions, and the key learnings are presented in Table  2. Importantly, cohesion in the research approach should help promote coordination of interventions and help realize the One Health approach to AMS in Australia. Building on and embedding health services research using implementation science frameworks should be a priority as this will support effective, sustainable and scalable implementation of AMS programmes.

**Table 2.** A summary of the key learnings from NCAS research

	Key learnings
1.	The widespread adoption of an audit platform to support **assessment of antimicrobial prescribing appropriateness** has facilitated monitoring of programmes and targets for intervention.
2.	**Rural and regional hospitals** need AMS models that boost access to local or network expertise and resources, and integration between acute, primary, aged and veterinary care. Telehealth models are successful.
3.	**SAP** remains the most common indication for antimicrobial use in Australian hospitals, with poor rates of appropriateness overall across all surgical specialties. SAP, therefore, remains a critical target for AMS, Australia-wide. Qualitative research identified several barriers related to surgeons’ priorities, prescriber autonomy, professional hierarchy and communication. Linking appropriateness to patient outcomes and providing benchmarking were identified as enablers.
4.	**AMS in the community** requires major investment. There are many structural factors relating to GP prescribing systems and access to guidelines and decision support; sociocultural factors influencing patient and physician preferences; and a lack of clear leadership in the sector. Opportunities for collaboration between GPs and community pharmacists for AMS programmes were identified.
5.	**The Aged Care NAPS** has identified major gaps in prescribing quality. The research highlighted important opportunities for nurses to take an active role in AMS and end-of-life antibiotic decision-making. Provision of education and clinical pathways, and improved documentation of prescribing are key targets for action.
6.	Several survey-based studies contributed new information about the **appropriateness of prescribing in companion animal veterinary practices** and highlighted the lack of AMS programmes. Unique issues that veterinarians face include the cost of microbiological testing, client expectations, a lack of industry-independent prescribing guidelines and a lack of financial incentives for AMS programmes. There was, however, a willingness to change practices and widespread recognition that appropriate use of antimicrobials was critical to human and animal health.
7.	In **livestock production**, Australia has very limited usage of antimicrobials of high importance. However, we still do not have a clear idea of the actual end-usage of antimicrobials in production animals. A mixed-methods study of in-water antimicrobial dosing on pig farms highlighted variations in how antimicrobial regimens are calculated, prepared and delivered, and identified knowledge gaps among farm managers.
8.	**Lack of education and training** and resources for AMS was identified as a consistent theme across all areas.
